# Large‐Scale Genotype‐Based Trait Imputation With Multi‐Ancestry GWAS Data

**DOI:** 10.1002/gepi.70030

**Published:** 2026-01-15

**Authors:** Jingchen Ren, Wei Pan

**Affiliations:** ^1^ School of Statistics University of Minnesota Minneapolis Minnesota USA; ^2^ Division of Biostatistics and Health Data Science, School of Public Health University of Minnesota Minneapolis Minnesota USA

**Keywords:** Alzheimer's disease, GWAS summary data, LS‐imputation, SNP, transfer learning

## Abstract

Genome‐wide association studies (GWAS) have been instrumental in identifying genetic variants associated with complex traits and diseases, including Alzheimer's disease (AD). However, traditional GWAS approaches often focus on European populations, which may lead to loss of power and limit the generalizability of findings across diverse ancestries. On the other hand, LS‐Imputation, a nonparametric trait imputation method, leverages GWAS summary statistics and genotype data to impute missing traits, which can then be used for GWAS and other downstream analyses. Although LS‐Imputation has been applied successfully to European populations, its performance in non‐European populations would be hindered by smaller sample sizes, leading to reduced imputation accuracy. To address these limitations, we propose two novel variants of LS‐Imputation‐LS‐Imputation‐Combined and LS‐Imputation‐Transfer—designed to integrate multi‐ancestry GWAS data and enhance imputation performance. LS‐Imputation‐Combined optimally combines GWAS summary statistics from multiple ancestries, while LS‐Imputation‐Transfer sequentially refines imputed trait values across ancestries using stochastic gradient descent. We evaluate these methods using data from the UK Biobank and the Alzheimer's Disease Sequencing Project (ADSP), first applying them to high‐density lipoprotein (HDL) cholesterol levels as a proof‐of‐concept before focusing on imputing AD status in Black individuals for genetic association analysis. Our results demonstrate that integrating multi‐ancestry GWAS data improves trait imputation accuracy, with LS‐Imputation‐Transfer achieving the highest performance.

## 1 Introduction

Genome‐wide association studies (GWAS) have been instrumental in identifying genetic variants associated with complex traits and common diseases over the past few years (Abdellaoui et al. 2023). Alzheimer's disease (AD), one of the most common causes of dementia and the most prevalent multifactorial neurodegenerative disease (Hardy and Selkoe 2002; Prince 2013), has garnered significant attention in genetic research due to its high heritability, estimated to be between 60% and 80% (Gatz 2006). A key challenge in AD research is its late onset, with diagnosis typically occurring in individuals over 65 (DeTure and Dickson 2019), making it difficult to assess AD risk in younger populations.

With the rapid expansion of available AD GWAS data (Bellenguez et al. 2022; Kunkle et al. 2019; Wightman et al. 2021), there is a significant opportunity to develop novel methods for early AD risk assessment. Recently, LS‐Imputation, a nonparametric method, has been proposed as a potential solution, requiring only GWAS summary statistics and individual genotype data (Ren, Lin, He, et al. 2023). However, like many previous AD studies (Bellenguez et al. 2022; Kunkle et al. 2019; Schwartzentruber et al. 2021), LS‐Imputation has primarily focused on European populations. Although the method can be readily applied to other ancestry groups, the typically and relatively small sample sizes of non‐European ancestries available even in large‐scale biobanks (e.g., UK Biobank [UKB]; Bycroft et al. 2018) limit its imputation accuracy for minority populations.

Previous studies have demonstrated that trans‐ethnic meta‐analyses of GWAS data can enhance statistical power and facilitate the identification of additional risk variants (Wojcik et al. 2019), and numerous susceptibility loci for AD have been reported, particularly in American and European populations (Jun 2017; Kunkle et al. 2021). To leverage multi‐ancestry GWAS data and improve trait imputation performance, we propose two novel extensions of LS‐Imputation: LS‐Imputation‐Combined and LS‐Imputation‐Transfer. We evaluate these methods using data from the UKB and the Alzheimer's Disease Sequencing Project (ADSP), assessing their performance in imputing high‐density lipoprotein (HDL) levels and AD risk among Black individuals. Applied to HDL levels and AD in Black individuals using UKB and ADSP data, LS‐Imputation‐Transfer generally outperformed LS‐Imputation‐Combined. The best imputation results were achieved when using White ancestry GWAS as the initial values, with parameter tuning playing a crucial role. Multi‐ancestry imputation improved imputation accuracy and downstream association analysis. The study highlights the importance of leveraging multi‐ancestry genetic data for improving the performance of trait imputation and downstream analysis.

## 2 Materials and Methods

### 2.1 Review: LS‐Imputation With Single‐Ancestry Data

Suppose we have a GWAS summary data set $\left\{{\hat{\beta }}_{j}^{*}:j=1,\ldots ,p\right\}$ for a quantitative trait with p SNPs derived from an individual‐level GWAS data set $\left(X^{*},Y^{*}\right)$ of a single‐ancestry; given a new individual‐level data set of genotypes X with sample size $n_{2}$, we aim to impute the missing trait values Y for these $n_{2}$ individuals. The basic idea for the LS‐Imputation method is simple: Suppose X is standardized to have column mean 0 and variance 1; if Y were observed, we could compute the marginal association estimates as $\hat{\beta }=X^{\prime }Y∕\left(n_{2}-1\right)$. Thus, if we had $\hat{\beta }$ instead of Y, we could obtain Y by solving the system of equations $\hat{\beta }=X^{\prime }Y∕\left(n_{2}-1\right)$ (assuming $p>>n_{2}$). In reality, we may not have $\hat{\beta }$, but ${\hat{\beta }}^{*}$ could be used to approximate $\hat{\beta }$; as $X^{*}$ and X come from the same population, the corresponding marginal association estimates ${\hat{\beta }}^{*}$ and $\hat{\beta }$ should be close. We can now impute the trait values as follows:

(1) $$\hat{Y}=\text{arg}\min_{Y}{∥{\hat{\beta }}^{*}-\frac{1}{n_{2}-1}X^{\prime }Y∥}^{2}=\left(n_{2}-1\right){\left(XX^{\prime }\right)}^{+}X{\hat{\beta }}^{*}=\left(n_{2}-1\right)X^{\prime +}{\hat{\beta }}^{*},$$

where $A^{+}$ is the Moore–Penrose generalized inverse for matrix A; in practice, $A^{+}$ could be approximated by $A^{+}\approx {\left(A^{\prime }A+\lambda I\right)}^{-1}A^{\prime }$ with $\lambda =10^{-6}$ because $A^{+}=\lim_{\lambda \to 0^{+}}{\left(A^{\prime }A+\lambda I\right)}^{-1}A^{\prime }$. In practice, as discussed in Ren, Lin, He, et al. (2023) and Ren and Pan (2024), we may split the genotype matrix X into several smaller batches and select a subset of the SNPs (most of which are the more significant ones) to perform the imputation, ensuring computational feasibility.

The LS‐Imputation method is rooted in ordinary least squares (OLS) for quantitative traits, where GWAS summary statistics are derived from a linear regression model for each SNP. However, for binary traits such as AD status, GWAS summary statistics are typically obtained using a generalized linear model (GLM), such as logistic regression. To adapt the LS‐Imputation method for binary traits, we apply an approximation formula (Knutson et al. 2020) to convert GLM‐based summary statistics into linear model (LM)‐based summary statistics:

(2) $$\beta_{1}=\frac{e^{-b_{0}}}{{\left(1+e^{-b_{0}}\right)}^{2}}b_{1}$$

where $b_{0}$ is the (estimated) log odds of being a case for the SNP reference group, which can be estimated as the log ratio of the numbers of cases and controls; $b_{1}$ is the (estimated) log odds ratio for the SNP given the logistic regression‐based GWAS summary data. After obtaining the LM‐based summary statistics, we can apply LS‐Imputation as before.

### 2.2 New Methods With Multi‐Ancestry Data

Now we consider the problem with multi‐ancestry data. To be concrete, we consider two ancestries. Suppose that we have two GWAS summary datasets $\left\{{\hat{\tilde{\beta }}}_{1}^{*}\right\}$ and $\left\{{\hat{\tilde{\beta }}}_{2}^{*}\right\}$ from two ancestries, and a genotype matrix X from the second ancestry. Our goal is to impute Y for the second ancestry. Often, the sample size of the GWAS summary data for the second ancestry is much smaller than that from the first ancestry. We aim to maximize the performance by borrowing information from the GWAS summary data from the first ancestry.

#### 2.2.1 LS‐Imputation‐Combined

To integrate GWAS data from multiple ancestries into LS‐Imputation, we propose the following optimization problem:

(3) $$\hat{Y}=\text{arg}\min_{Y}\omega {∥{\hat{\tilde{\beta }}}_{1}^{*}-\frac{1}{n_{2}-1}X^{\prime }Y∥}^{2}+\left(1-\omega \right){∥{\hat{\tilde{\beta }}}_{2}^{*}-\frac{1}{n_{2}-1}X^{\prime }Y∥}^{2}$$

where ${\hat{\tilde{\beta }}}_{1}^{*}$ and ${\hat{\tilde{\beta }}}_{2}^{*}$ are the marginal effect sizes obtained from two different GWAS data sets of possibly different ancestries. Note that the significant SNPs selected for imputation from the different GWAS data sets may not be the same, so the dimensions of ${\hat{\tilde{\beta }}}_{1}^{*}$ ($p_{1}\times 1$) and ${\hat{\tilde{\beta }}}_{2}^{*}$ ($p_{2}\times 1$) may differ. In practice, we may use $\tilde{X}$ and $\tilde{\tilde{X}}$ to do the imputation, where $\tilde{X}$ ($n_{2}*p_{1}$) and $\tilde{\tilde{X}}$ ($n_{2}*p_{2}$) are both subsets of X. The selection of ω could be performed on a validation set; notice that when ω=0 or ω=1, the above method degenerates to the case where only the GWAS summary statistics from one ancestry are used for imputation. The above optimization problem has a closed‐form solution that can be used in practice:

(4) $$\hat{Y}=\left(n_{2}-1\right){\left(\omega \tilde{X}{\tilde{X}}^{\prime }+\left(1-\omega \right)\tilde{\tilde{X}}{\tilde{\tilde{X}}}^{\prime }\right)}^{+}\left(\omega \tilde{X}{\hat{\tilde{\beta }}}_{1}^{*}+\left(1-\omega \right)\tilde{\tilde{X}}{\hat{\tilde{\beta }}}_{2}^{*}\right),$$

#### 2.2.2 LS‐Imputation‐Transfer

Another variant of the LS‐Imputation method to integrate GWAS data from different ancestries is as follows. Consider the optimization problem:

(5) $$\hat{Y}=\text{arg}\min_{Y}{∥{\hat{\tilde{\beta }}}_{2}^{*}-\frac{1}{n_{2}-1}\tilde{\tilde{X}}Y∥}^{2},$$

where ${\hat{\tilde{\beta }}}_{2}^{*}$ represents the marginal effect size obtained from the second ancestry, the target population for which we aim to impute Y with the corresponding genotype data $\tilde{\tilde{X}}$. We use $\hat{Y}$, imputed with the GWAS summary statistics from the first ancestry, as the initial value, and then we apply SGD to solve the above optimization problem.

In this method, we initialize the estimation procedure using results from an auxiliary data set of a different ancestry (e.g., European) and iteratively refine the estimates using stochastic gradient descent (SGD) to better align with the target data—specifically, the ancestry‐specific summary statistics. This approach is a form of transfer learning, a concept originating from and widely used in deep learning to leverage knowledge from related tasks or domains (Pan and Yang 2010). Due to its strong empirical performance, transfer learning has seen broad adoption in practice. However, there is a key distinction. In deep learning, where the objective function is typically non‐convex, different initializations often lead to different local optima. A validation set is then used to select the best‐performing solution. In contrast, our objective function is quadratic and convex; thus, if SGD is run to full convergence, it will converge to the unique global optimum—equivalent to the solution obtained by LS‐Imputation using only the target ancestry GWAS. In this case, the informative initialization from the auxiliary data would be effectively discarded. To preserve the benefits of this initialization, we introduce early stopping, selecting the optimal number of SGD iterations using a validation data set. As in deep learning, we employ SGD not only for its computational efficiency but also for its strong empirical performance in large‐scale data settings (Bottou 2010).

### 2.3 UKB: HDL and AD

As a proof of concept, we selected the HDL trait from the UKB due to its large sample size of White British ancestry and a much smaller sample size of Black ancestry, reflecting realistic study scenarios. Although all trait values were known, we treated a subset of Black ancestry values as missing, allowing us to impute them and compare the imputed values with observed ones to evaluate method performance.

We first conducted a GWAS based on 178,175 individuals of White British ancestry. We then extracted self‐reported Black/Black British individuals (including Caribbean, African, and other Black backgrounds) using UKB data field 21000, codes 4001, 4002, and 4003. To ensure data completeness, we included only individuals with no missing HDL measurements, resulting in a final data set of 6607 individuals. These individuals were randomly split into three groups: 3304 (50%) for training to obtain the HDL GWAS, 1321 (20%) for validation to select tuning parameters, and 1982 (30%) for testing. For SNP filtering, we excluded variants with a minor allele frequency (MAF) <0.05, those with >10% missing values, and those failing the Hardy–Weinberg equilibrium (HWE) exact test (*p* value <0.001). Additionally, we applied linkage disequilibrium (LD) pruning using a window size of 50, a step size of 5, and an $r^{2}$ threshold of 0.8, resulting in a final set of 517,115 SNPs.

When imputing the HDL trait using GWAS summary statistics from the White ancestry cohort (178,175 individuals), we used either 20,000 or 30,000 SNPs. Among these, 19,069 SNPs had *p* values <0.05 in the training data, while the remaining 931 or 10,931 SNPs were randomly selected from nonsignificant variants in the training set.

For imputation using GWAS summary statistics from the Black ancestry cohort (3304 individuals), we selected either 30,000 or 50,000 SNPs. When using 30,000 SNPs, we randomly selected them from the 30,813 significant SNPs (*p* value <0.05) in the training data. When using 50,000 SNPs, we included all 30,813 significant SNPs, with an additional 19,187 SNPs randomly selected from the remaining variants.

For AD, we extracted self‐reported Black/Black British individuals (including Caribbean, African, and other Black backgrounds), as well as White/Black African and White/Black Caribbean individuals, using UKB data field 21000, codes 2001, 2002, 4001, 4002, and 4003. This selection resulted in a total of 8608 individuals. We applied the same quality control (QC) procedures for SNP filtering as described previously. After intersecting the filtered SNPs with the AD GWAS data set, we obtained 473,030 SNPs for analysis.

For European ancestry AD GWAS data, we identified 36,186 SNPs with *p* values <0.05. All these SNPs were included for imputation, along with 3814 additional randomly selected SNPs, resulting in a total of 40,000 SNPs.

For African American AD GWAS data, we identified 34,345 SNPs with *p* values <0.05. We then randomly selected 5655 additional SNPs from the remaining variants, yielding a total of 40,000 SNPs for imputation.

### 2.4 Testing With the ADSP Data

The ADSP aims to identify genetic variants associated with AD susceptibility and progression through large‐scale genomic sequencing studies of individuals affected by AD and cognitively normal controls. For this study, we used Release 4 of the ADSP whole‐genome sequencing (WGS) data. Following QC procedures for both phenotype and genotype, as recommended by ADSP, we obtained a data set of 33,143 individuals.

For the purpose of evaluation, we focused on individuals of Black or African American ancestry, resulting in a subset of 5567 individuals, which was further divided into 2783 individuals for validation and 2784 individuals for testing. We used the validation data to select the tuning parameters for the imputation methods, then applied them to the test data. The genotype data underwent the same QC procedures as the UKB data set. After further intersecting the filtered SNPs with those available in the AD GWAS data set, we obtained a final set of 377,092 SNPs for analysis.

For European ancestry AD GWAS data (as discussed in the next subsection), we identified 28,618 SNPs with *p* values <0.05. All of these SNPs were included for imputation, along with 1,382 additional randomly selected SNPs, resulting in a total of 30,000 SNPs.

For African American AD GWAS data (in the next subsection), we identified 27,773 SNPs with *p* values <0.05. To reach a total of 30,000 SNPs, we randomly selected 2227 additional SNPs from the remaining variants.

### 2.5 AD GWAS Data

For the AD GWAS summary data, we utilized a published data set (Lake et al. 2023), including GWAS results from both European ancestry and African American ancestry cohorts. The European ancestry cohort consists of 85,934 cases (including 39,106 diagnosed cases and 46,828 proxy cases) and 401,577 controls. The African American ancestry cohort includes 2748 cases and 5222 controls. To implement the LS‐Imputation method, all GWAS summary statistics were harmonized to match the reference alleles of the corresponding ADSP or UKB data.

## 3 Results

### 3.1 Proof‐of‐Concept: HDL

**Figure 1:**
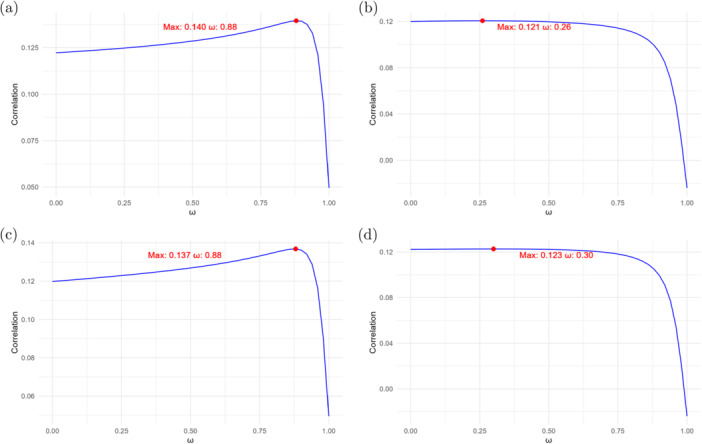
Comparison of the correlation between observed and imputed HDL trait values on validation data: (a) 20,000 SNPs from Black ancestry and 50,000 SNPs from White ancestry; (b) 30,000 SNPs from Black ancestry and 30,000 SNPs from White ancestry; (c) 20,000 SNPs from Black ancestry and 30,000 SNPs from White ancestry; and (d) 30,000 SNPs from Black ancestry and 50,000 SNPs from White ancestry.

Figure 1 illustrates how the correlation changes with ω for different combinations of the number of SNPs used during imputation on the validation data for Black individuals in UKB. When ω=0, only Black ancestry GWAS summary statistics were used for imputation, whereas when ω=1, only the White ancestry GWAS summary data were used. The results indicate that using only either Black or White ancestry GWAS summary statistics for imputation does not yield optimal performance. Among all tested parameter combinations, the highest correlation (0.140) was achieved when $\tilde{X}=1,321\times 20,000,\tilde{\tilde{X}}=1,321\times 50,000$, and ω=0.88. This set of parameters will be used in the subsequent analyses.

Table 1 presents the correlation between observed HDL trait values and LS‐Imputation‐Transfer imputed trait values on the validation data for Black individuals in the UKB. The maximum correlation achieved here is slightly higher than that obtained with LS‐Imputation‐Combined. We observed that, compared to the learning rate, the initial value derived from LS‐Imputation using White ancestry GWAS summary statistics has a more significant impact on the correlation. For different learning rates, the max correlation we could obtain for different initial values is almost the same, which implies that our method is robust to the learning rate. The optimal setting involves using the initial value obtained from LS‐Imputation with White ancestry GWAS summary statistics with 20,000 SNPs, a learning rate of 0.1, and 10 epochs. This configuration could be applied to the imputation on the test data for the LS‐Imputation‐Transfer method; however, in order to confirm the robustness of our proposed method, the results with two other different learning rates are shown next.

**Table 1:** Correlation comparison for different parameters in HDL imputation for Black individuals in the UK Biobank using LS‐Imputation‐Transfer.

# of SNPs (initial value)	LR	# of epochs (Max correlation)	Max correlation
20,000	0.1	10	0.1631
30,000	0.1	16	0.1485
20,000	0.01	103	0.1629
30,000	0.01	168	0.1482
20,000	0.001	1027	0.1629
30,000	0.001	1685	0.1481

The correlations between the observed and imputed trait values on the test data are 0.110 and 0.140 for the LS‐Imputation‐Combined and LS‐Imputation‐Transfer methods, respectively. The correlation on the test data is only slightly lower than that obtained on the validation data. Notably, the LS‐Imputation‐Transfer result here is derived using the best setting from the validation data set. Since the true HDL values are known, we can also apply LS‐Imputation‐Transfer directly to the test data to determine whether it achieves the maximum correlation. Table S1 presents the correlation values for different parameter settings in HDL imputation for Black individuals in the UKB test data set. When using the best parameters identified from the validation data, for all three different learning rates, the correlations are around 0.1404—slightly lower than the highest achievable correlation on the test data, which is around 0.1480. As the correlations obtained with different learning rates are almost the same, we confirm that our method is not sensitive to the choice of learning rate.

**Figure 2:**
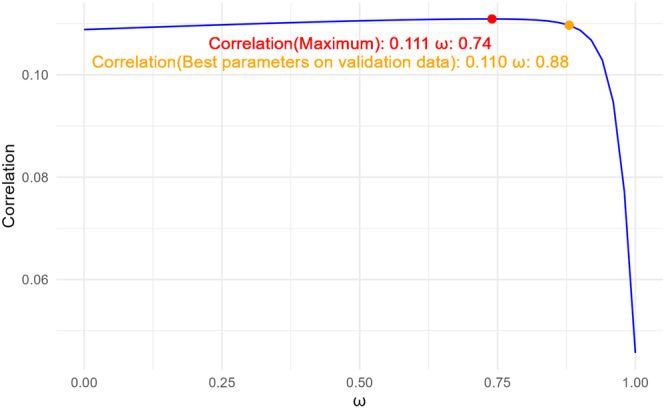
Varying correlations with ω for LS‐Imputation‐Combined on the test data for HDL in the UK Biobank.

**Figure 3:**
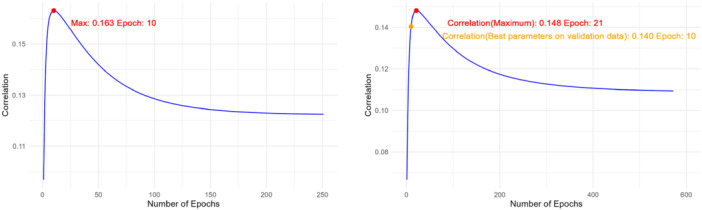
Varying correlations with the number of epochs for LS‐Imputation‐Transfer. Left: Validation data; Right: Test data.

Figure 2 illustrates how the correlation varies with ω on the test data. The correlation (0.110) obtained using the best parameters from the validation data is very close to the maximum correlation (0.111) achievable on the test data. Similarly, Figure 3 demonstrates how the correlation changes with the number of epochs on both the validation and test data sets. In both cases, the correlation initially increases and then decreases. Importantly, when applying the best validation parameters to the test data, the resulting correlation is very close to the highest achievable one.

### 3.2 Evaluation of AD Imputation With the ADSP Data

We applied the imputation methods, along with the AD GWAS data, to the ADSP validation and test data, respectively, to impute the AD status. The observed AD status in the validation data was known and used to select optimal tuning parameters, which were then applied to impute AD status in the test data set. Finally, we compared the observed and imputed AD statuses in the test data set to assess the performance of the imputation methods.

**Figure 4:**
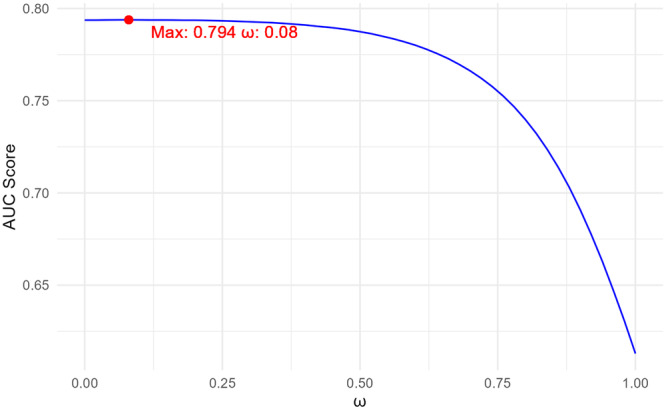
AUC varying with ω for LS‐Imputation‐Combined on the validation data for ADSP.

Figure 4 illustrates how the AUC score varies with ω on the validation data for ADSP. Similar to the previous observations, when ω=0, only Black ancestry GWAS summary statistics are used for imputation, whereas when ω=1, only European ancestry GWAS summary statistics are utilized. The highest AUC score achieved is 0.794 at ω=0.08. Using only European ancestry GWAS summary statistics for imputation in Black/African American individuals results in the lowest AUC of 0.613, while using only Black/African ancestry GWAS summary statistics performs well, achieving an AUC of 0.7937, very close to the optimal AUC.

**Figure 5:**
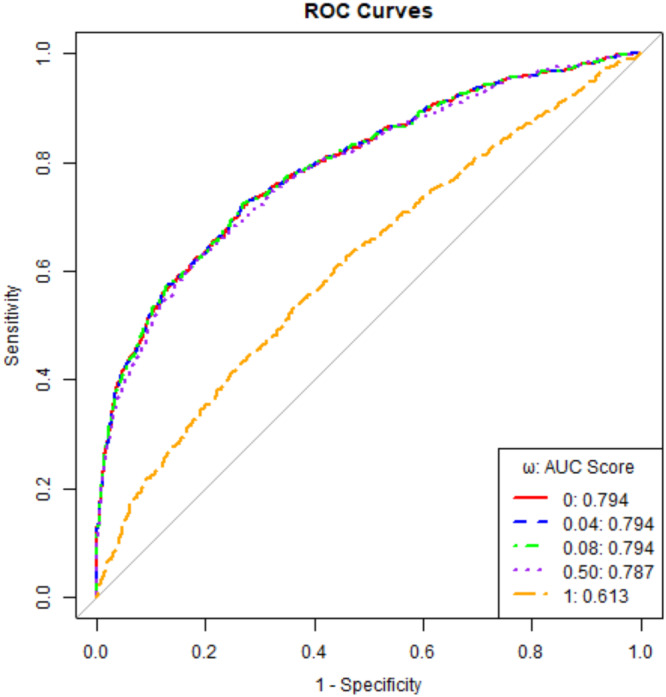
ROC curve for LS‐Imputation‐Combined on the validation data.

Figure 5 presents the ROC curves on the validation data for different ω values in LS‐Imputation‐Combined. It shows that ROC curves corresponding to similar AUC scores are nearly identical.

**Figure 6:**
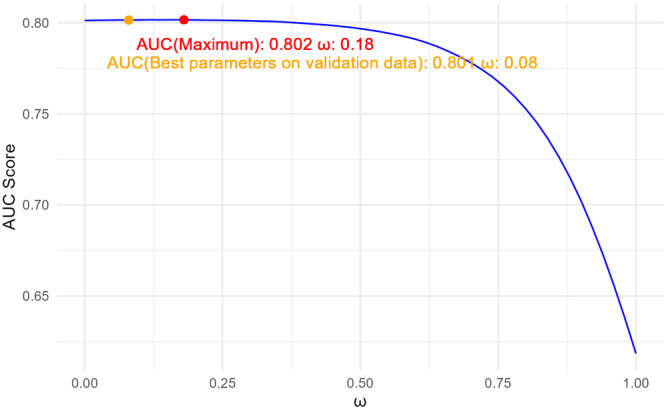
AUC varying with ω for LS‐Imputation‐Combined on the test data for ADSP.

Figure 6 depicts how the AUC score changes with ω on the test data. The AUC score obtained using the best parameter from the validation data is 0.801, which is very close to the highest achievable AUC score of 0.802 on the test data. This further confirms that applying the optimal parameters identified from the validation data to the test data is a reasonable approach.

For the LS‐Imputation‐Transfer method, the maximum AUC obtained on the validation data is 0.80, similar to the result from the LS‐Imputation‐Combined method. The learning rate used is 0.01, and the epoch number is 6069, at which the highest AUC was achieved. These parameters will be applied to the test data.

**Figure 7:**
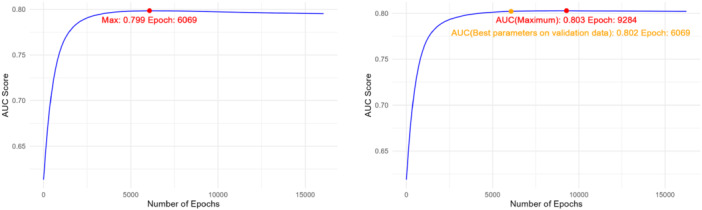
AUC varying with the number of epochs for LS‐Imputation‐Transfer. Left: Validation data; Right: Test data.

As we know the true AD status, we can also apply LS‐Imputation‐Transfer to the test data to check if it reaches the maximum correlation. Figure 7 shows how the AUC score changes with the number of epochs on both the validation and test data. We can observe that with the best parameters obtained from the validation data, the AUC on the test data is 0.802, while the maximum AUC achievable is 0.803. This confirms that using the optimal parameters from the validation data for imputation on the test data is a reasonable approach.

To further demonstrate that the multi‐ancestry imputation method has better performance, we conduct DeLong's test to compare the AUC for either LS‐Imputation‐Combined or LS‐Imputation‐Transfer (with selected best tuning parameters) with that from using only the data of European ancestry to impute. The *p* values for both methods are less than $2.2\times 10^{-16}$, confirming statistically significant performance differences of the new methods from using only European ancestry. Table 2 summarizes the performance of different imputation methods on the test data. We could see that for both traits, utilizing multi‐ancestry data to do imputation performs better than using only European‐ancestry data.

**Table 2:** Performance of different imputation methods. For LS‐Imputation‐Combined (multi‐ancestry) and LS‐Imputation‐Transfer, the results are based on the tuning parameters selected with the validation data.

Trait	Test data	Method	Measurement	Value
HDL	UKB	LS‐Imputation‐Transfer	Correlation	0.140
HDL	UKB	LS‐Imputation‐Combined (European only)	Correlation	0.046
HDL	UKB	LS‐Imputation‐Combined (multi‐ancestry)	Correlation	0.110
AD	UKB	LS‐Imputation‐Transfer	AUC	0.802
AD	UKB	LS‐Imputation‐Combined (European only)	AUC	0.619
AD	UKB	LS‐Imputation‐Combined (multi‐ancestry)	AUC	0.801

### 3.3 Application to the UKB: AD Imputation and Its Genetic Association Analysis

Finally, we applied the proposed methods to impute AD status for the Black cohort in the UKB. Due to the late onset nature of AD and relatively young participants in the UKB, there were only a few diagnosed AD cases. Hence, we would like to impute the AD status, that is, the genetic risk of AD, for 8608 Black individuals in the UKB. The application was similar to that for the ADSP data. In particular, we utilized the best parameter settings obtained from the ADSP validation data set for imputation here. For LS‐Imputation‐Combined, we selected ω=0.08, and for LS‐Imputation‐Transfer, the learning rate was set to 0.01 with 6069 epochs.

**Figure 8:**
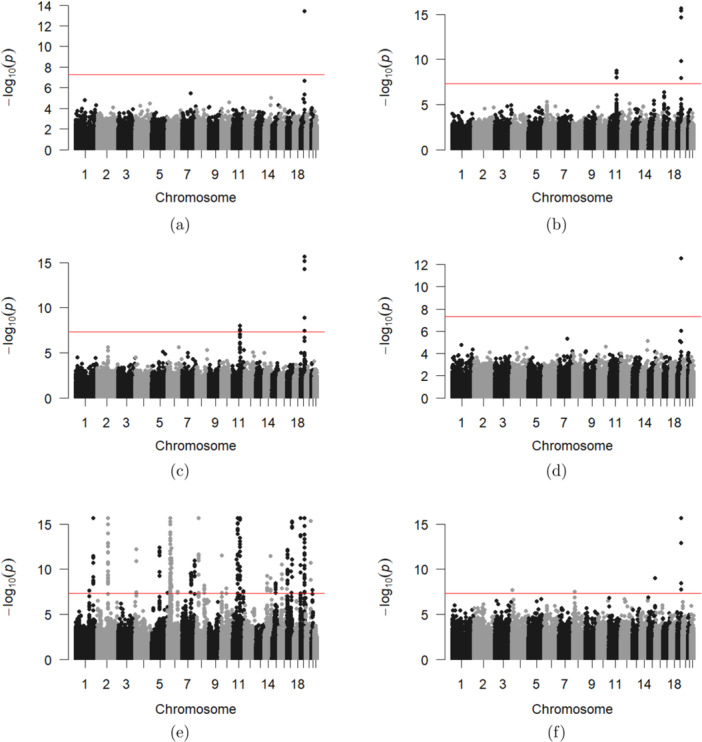
Manhattan plots of the UKB GWAS of Black individuals with AD status: (a) Imputed using LS‐Imputation‐Combined; (b) imputed using LS‐Imputation‐Transfer; (c) imputed using LS‐Imputation with the GWAS of White individuals; (d) imputed using LS‐Imputation with the GWAS of Black individuals; (e) Manhattan plot of the EADB GWAS for White individuals (used in LS‐Imputation); and (f) Manhattan plot of the ADSP GWAS for Black individuals. The red line corresponds to *p* value $5\times 10^{-8}$.

We conducted GWAS on the Black individuals with their AD statuses imputed by various methods. Figure 8 summarizes the GWAS results as the Manhattan plots. Although the sample size is relatively small, performing a GWAS using the AD status imputed by LS‐Imputation‐Transfer methods identifies several significant SNPs on chromosomes 19 and 11, while using LS‐Imputation‐Combined identifies only one significant SNP, rs429358 (Chr19:45411941 on Assembly GRCh37, *p* value < 2.2 × 10^−16^), located in the well‐established APOE locus, also identified by LS‐Imputation‐Transfer. In contrast, conducting a GWAS based on diagnosed AD cases or proxy‐AD cases does not detect any significant SNPs (not shown).

For comparison, Figure 8 also shows the Manhattan plots for LS‐Imputation using the GWAS summary statistics from either Black or White ancestry only. We observe that LS‐Imputation‐Transfer outperforms LS‐Imputation, which, using the GWAS summary statistics solely from Black ancestry, identifies only one significant SNP on chromosome 19 (the same rs429358 as above). On the other hand, when using only the GWAS summary statistics from White ancestry, we detect multiple significant SNPs on chromosomes 19 and 11, largely overlapping with those obtained by using LS‐Imputation‐Transfer. A detailed summary of all the significant SNPs identified with different LS‐Imputation methods can be found in Table S2. Many of the variants are in the APOE‐TOMM40 region on chromosome 19, including rs429358, rs2075650, rs6857, rs769450, rs12721051, and rs4420638. This region is the most well‐established genetic risk locus for late‐onset AD. In addition, we observe a strong signal on chromosome 11 near the PICALM locus (rs10792831), which is another locus consistently implicated in AD risk across populations.

We aimed to further confirm whether the significant SNPs identified by our proposed method are true signals. Since we did not have the *p* values for the GWAS summary statistics of Black ancestry used during our imputation method, we used GWAS summary statistics obtained from the ADSP Black individuals as a substitute. Figure 8 further compares the GWAS results obtained using either EADB (the GWAS summary statistics for White ancestry used in the imputation) or ADSP (Black individuals) with those derived from AD status imputed by LS‐Imputation‐Transfer. We observe that the significant SNPs identified by LS‐Imputation‐Transfer on chromosome 19 appear in both the EADB and ADSP Black ancestry GWAS. However, the significant SNPs on chromosome 11 are not detected in the ADSP Black ancestry GWAS, possibly due to its smaller sample size of only 5567 individuals. If the signal on chromosome 11 is confirmed for the Black ancestry, it would further support that leveraging data from different ancestries provides valuable benefits for downstream analysis with enhanced imputation performance.

Another application to binary trait hypertension with UKB data could be found in the Supplementary; similar conclusions to those for trait AD can be drawn.

## 4 Discussion

We have proposed two variants of the LS‐Imputation method, namely LS‐Imputation‐Transfer and LS‐Imputation‐Combined, to incorporate multi‐ancestry GWAS for LS‐Imputation. The basic idea is to borrow information for a smaller racial/ethnic group from the GWAS data of a larger group, which is quite general and widely applicable. For example, it has been shown to be useful in constructing polygenic scores (PGS) across ancestries (Zhang et al. 2024; Zhao et al. 2022). We have also demonstrated its effectiveness here with our proposed methods using HDL trait data from the UKB, as well as AD data from both the ADSP and UKB. Our results show that the imputation performance using multi‐ancestry GWAS is no worse than using a single ancestry for imputation in Black individuals.

Moreover, integrating GWAS data across diverse ancestries offers significant benefits, including increased statistical power in downstream analyses and enhanced generalizability of genetic discoveries. However, this approach comes with limitations and relies on important assumptions. A key assumption is that the genetic architecture of complex traits is at least partially shared across populations. In practice, differences in LD patterns, allele frequencies, and effect sizes between ancestries can introduce heterogeneity, complicating interpretation and potentially leading to ancestry‐specific biases. Such biases may reduce imputation accuracy, generate spurious associations, or obscure signals that are unique to specific populations.

In our study, both the LS‐Imputation‐Combined and LS‐Imputation‐Transfer methods demonstrated improved performance by leveraging multi‐ancestry GWAS data. However, these approaches rely on the critical assumption that effect sizes are sufficiently aligned across populations to enable effective information sharing. Transferring summary statistics from a larger ancestry group—such as European ancestry—to a smaller group—such as Black or African American ancestry—can inadvertently introduce bias if the underlying genetic associations differ substantially between populations. While parameter tuning using ancestry‐matched validation sets can help mitigate this risk, it may not eliminate it entirely. Future applications and further evaluations, especially with data from larger sample sizes for non‐European ancestries, are warranted.

Another potential direction for future work is to incorporate linear mixed model (LMM)‐based GWAS summary statistics into our LS‐imputation framework. Many modern GWAS now employ LMMs to account for population structure and cryptic relatedness. However, marginal effect estimates derived from OLS (as assumed in LS‐imputation) can be biased in the presence of population stratification or relatedness, potentially inflating association statistics and mis‐weighting their contributions during the imputation step. Addressing this challenge presents an important avenue for future research. Besides, in our current proposed methods, the number of SNPs to be used during the imputation process is data‐driven and empirically selected. As pointed out previously (Ren, Lin, He, et al. 2023; Ren, Lin, and Pan 2023; Ren and Pan 2024), we require $p>n_{2}$, and we should include as many informative SNPs as it is computationally feasible (e.g., all SNPs with *p* value <0.05 in the GWAS summary data set). In addition, we observed that including some (but not too many) nonsignificant SNPs could help improve the imputation performance. While the imputation accuracy can be modestly influenced by the number of SNPs used (which in practice may be selected using different *p* value thresholds), our empirical results demonstrate that the differences are small and the method remains relatively robust across a range of reasonable numbers of SNPs. However, the relationship between the number of SNPs used in the imputation process and the chosen batch size is complex, for which we do not yet have a rigorous theoretical characterization. This would also be an interesting direction for future work.

## Supporting information

Suppl2.

## Data Availability

The AD GWAS summary data are publicly available at https://ndkp.hugeamp.org/dinspector.html?dataset=Lake2023_AD_Mixed. Individual‐level data from the UK Biobank (UKB, https://www.ukbiobank.ac.uk/) and the Alzheimer's Disease Sequencing Project (ADSP, https://adsp.niagads.org/) are available by application through their respective data access processes. The code used for the analyses presented in this paper is available at https://github.com/ren328/LS-Multi-Ancestry. The data that support the findings of this study are available from the UKB and ADSP. Restrictions apply to the availability of these data, which were used under license for this study. UKB data are available from https://www.ukbiobank.ac.uk/ with the permission of UKB; ADSP data are available from https://adsp.niagads.org/ with the permission of ADSP.
