# Supplementary material for: Large‐Scale Genotype‐Based Trait Imputation With Multi‐Ancestry GWAS Data
Source: Genet Epidemiol. 2026 Jan 15;50(1):e70030. doi: 10.1002/gepi.70030 (PMC12805644; doi:10.1002/gepi.70030)
Supplement: Supplementary file 1 — Suppl2. [file GEPI-50-0-s001.pdf]

Supplementary to “Large-scale genotype-based trait imputation with multi-ancestry GWAS data”

Jingchen Ren[1,2] and Wei Pan[2,*]

[1]School of Statistics, University of Minnesota, Minneapolis, MN, 55455

[2]Division of Biostatistics and Health Data Science, School of Public Health, University of Minnesota, Minneapolis, MN, 55455

[*]Corresponding author. Email: panxx014@umn.edu

Correlation comparison for different parameter settings in HDL imputation for Black individuals in the UK Biobank using LS-Imputation-Transfer

# of SNPs (Initial Value)	LR	# of epochs	Correlation
20,000	0.1	10	0.1404
20,000	0.1	21	0.1481
20,000	0.01	103	0.1404
20,000	0.01	218	0.1480
20,000	0.001	1027	0.1404
20,000	0.001	2185	0.1480

Table 1: Correlation comparison for different parameter settings in HDL imputation for Black individuals in the UK Biobank using LS-Imputation-Transfer.

Application on hypertension

We implemented our proposed method on another binary trait, hypertension, using UK Biobank data. Similar to the HDL case, we first obtained the hypertension GWAS based on 204,169 individuals of White British ancestry. We then extracted the self-reported Black/Black British individuals (including Caribbean, African, and any other Black backgrounds) based on data field 21000, codes 4001, 4002, and 4003. We considered individuals with no missing values in the hypertension data, resulting in 8,629 individuals being included in our study. Of these, 4,314 (50%) individuals were used as training data to obtain the GWAS for Black individuals; 1,727 (20%) individuals were used as validation data to select tuning parameters; and 2,588 (30%) individuals were used as test data.

We filtered out SNPs with a minor allele frequency less than 0.05, missing values greater than 10%, or those failing the Hardy-Weinberg equilibrium exact test with a p-value less than 0.001. Additionally, we pruned out SNPs in high linkage disequilibrium (LD) with a window size of 50, a step size of 5, and an r^2 threshold of 0.8, resulting in a final set of 497,965 SNPs.

For White ancestry, we identified 16,858 SNPs with a p-value less than 0.05 and included all of these SNPs, while randomly selecting 3,142 additional SNPs for imputation, bringing the total to 20,000 SNPs. For Black ancestry, we identified 46,346 SNPs with a p-value less than 0.05 and included all of them, then we randomly selected 3,654 SNPs from the remaining SNPs, resulting in a total of 50,000 SNPs used for imputation.

As shown in Figure 1, for the LS-Imputation-Combined method, the maximum AUC we could achieve is 0.558 with $\omega = 0.96$. Regarding the AUC on the test data, as shown in Figure 2, the maximum AUC we could reach is 0.561 when $\omega = 0.68$, while the AUC obtained with the best parameters on the validation data is 0.555, slightly lower than the maximum.

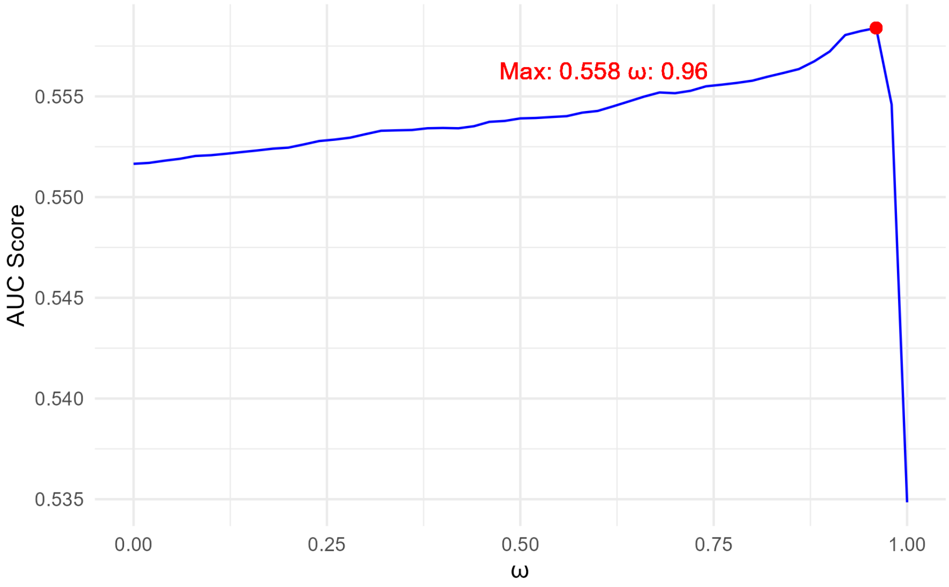

Figure 1: AUC varying with ω for LS-Imputation-Combined on the validation data for HPT in the UK Biobank.

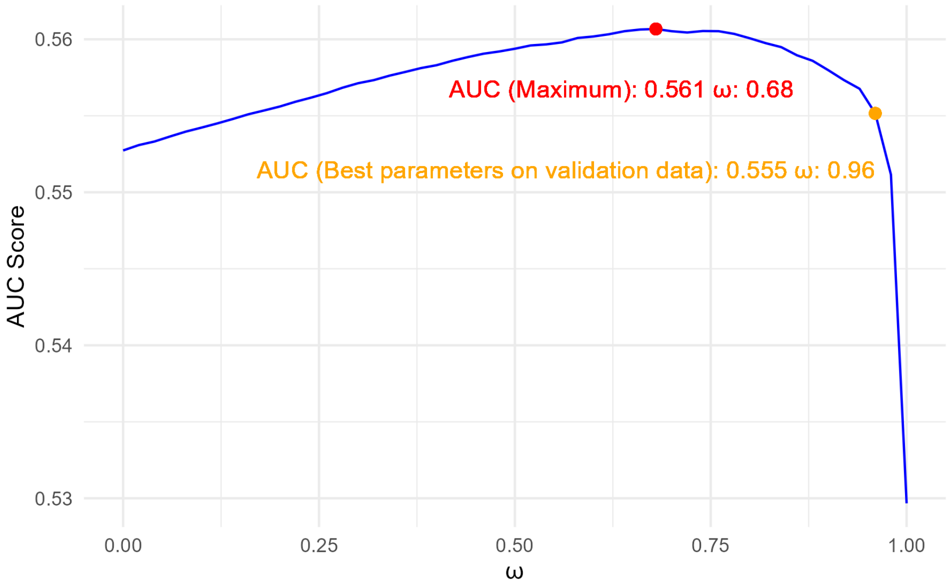

Figure 2: AUC varying with ω for LS-Imputation-Combined on the test data for HPT in the UK Biobank.

Figure 3 further demonstrates the ROC curves for the LS-Imputation-Combined method on the test data with different values of ω. We can see that using the LS-Imputation-Combined method outperformed using the GWAS of only one ancestry for

imputation.

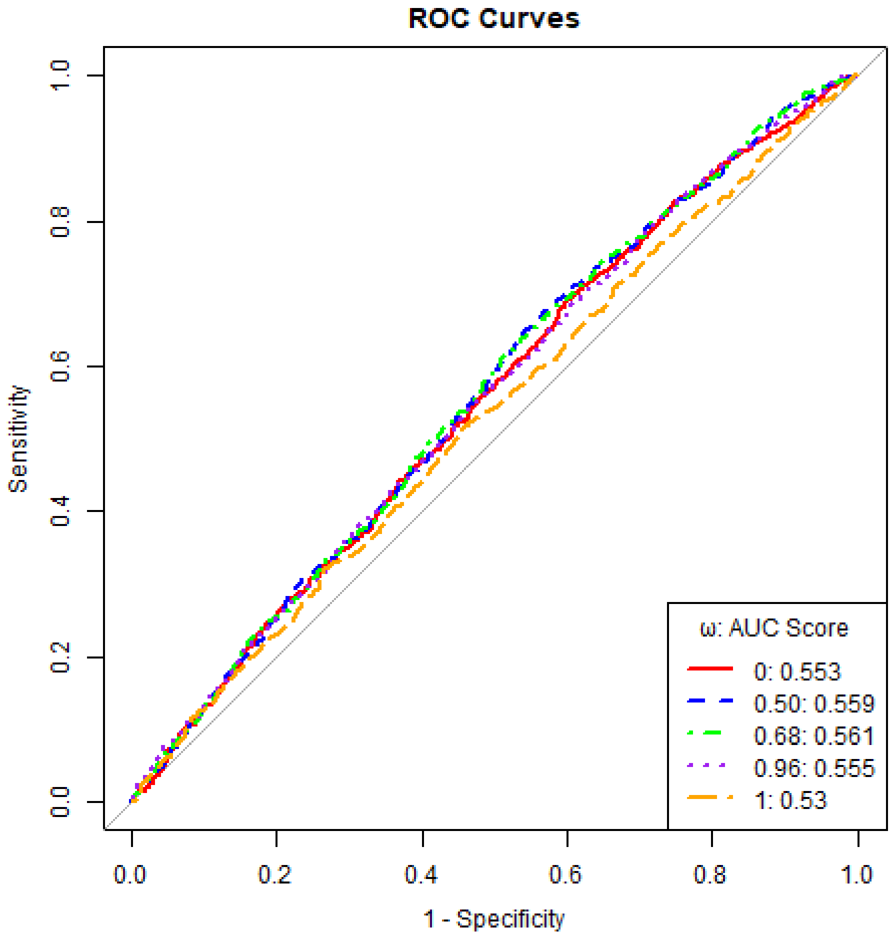

Figure 3: ROC curves for LS-Imputation-Combined on the test data.

For the LS-Imputation-Transfer method, the learning rate was set to 0.1. The maximum AUC on the validation data was 0.581, reached at epoch 14. On the test data, the maximum AUC we could achieve was 0.579 at epoch 16, the same as that obtained with the best parameter on the validation data. The LS-Imputation-Combined method reached a slightly higher AUC than that of LS-Imputation-Transfer.

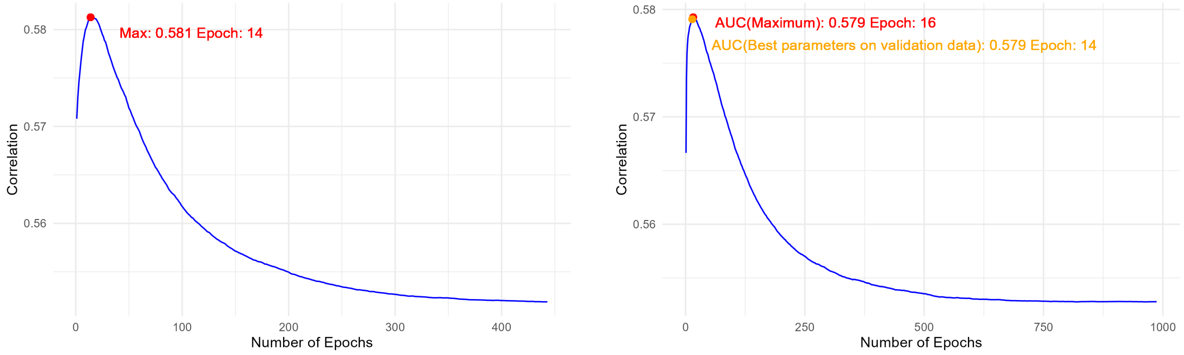

Figure 4: Correlation varying with the number of epochs for LS-Imputation-Transfer for hypertension in the UK Biobank. Left: Validation data; Right: Test data.

Summary of significant SNPs identified via different methods

SNP	CHR	Position	p-value	Identified Method
rs4944560	11	85845045	9.26×10^{-9}	1
rs3844143	11	85850243	1.71×10^{-9}	1
rs676733	11	85734010	9.20×10^{-9}	4
rs1898895	11	85837669	3.56×10^{-8}	4
rs10792831	11	85852979	2.94×10^{-9}	1,4
rs6857	19	45392254	3.92×10^{-16}	1,4
rs184017	19	45394969	2.08×10^{-15}	1,4
rs2075650	19	45395619	1.48×10^{-10}	1,4
rs34404554	19	45395909	1.12×10^{-8}	1,4
rs34878901	19	45402477	4.03×10^{-16}	1,4
rs769450	19	45410444	2.15×10^{-15}	1,4
rs429358	19	45411941	$<2.2\times10^{-16}$	1,2,3,4
rs12721051	19	45422160	$<2.2\times10^{-16}$	1,4
rs4420638	19	45422946	$<2.2\times10^{-16}$	1,4

Table 2: Summary of significant SNPs identified via different methods. Method 1: LS-Imputation-Transfer; Method 2: LS-Imputation-Combined (Multi-Ancestry); Method 3: LS-Imputation (Black Ancestry Only); Method 4: LS-Imputation (White Ancestry Only).
